# The Right to Move: A Multidisciplinary Lifespan Conceptual Framework

**DOI:** 10.1155/2012/873937

**Published:** 2012-12-03

**Authors:** Toni C. Antonucci, James A. Ashton-Miller, Jennifer Brant, Emily B. Falk, Jeffrey B. Halter, Levent Hamdemir, Sara H. Konrath, Joyce M. Lee, Wayne R. McCullough, Carol C. Persad, Roland Seydel, Jacqui Smith, Noah J. Webster

**Affiliations:** ^1^Institute for Social Research, University of Michigan, 426 Thompson St, Ann Arbor, MI 48106, USA; ^2^Department of Psychology, University of Michigan, 1012 East Hall, 530 Church St, Ann Arbor, MI 48109, USA; ^3^Department of Mechanical Engineering, University of Michigan, 3208 G. G. Brown Laboratory, 2350 Hayward, Ann Arbor, MI 48109, USA; ^4^Department of Internal Medicine, Division of Geriatric Medicine, University of Michigan, 300 North Ingalls, Ann Arbor, MI 48109, USA; ^5^NuStep, Inc., 5111 Venture Dr, Ann Arbor, MI 48108, USA; ^6^Department of Communication Studies, University of Michigan, 105 South State St, Ann Arbor, MI 48109, USA; ^7^Consumer and Market Insights, Amway, 187 Monroe Ave NW, Grand Rapids, MI 49503, USA; ^8^Division of Pediatric Endocrinology, Child Health Evaluation and Research Unit, University of Michigan, 300 NIB, Room 6E18, Campus Box 5456, Ann Arbor, MI 48109, USA; ^9^Department of Psychiatry, University of Michigan, 2101 Commonwealth Blvd Ste C, Ann Arbor, MI 48105, USA; ^10^University Center for the Development of Language and Literacy, University of Michigan, 1111 Catherine St, Ann Arbor, MI 48109, USA; ^11^Adidas Innovation Team, Adidas, 5055 N Greeley Ave, Portland, OR 97217, USA

## Abstract

This paper addresses the health problems and opportunities that society will face in 2030. We propose a proactive model to combat the trend towards declining levels of physical activity and increasing obesity. The model emphasizes the need to increase physical activity among individuals of all ages. We focus on the right to move and the benefits of physical activity. The paper introduces a seven-level model that includes cells, creature (individual), clan (family), community, corporation, country, and culture. At each level the model delineates how increased or decreased physical activity influences health and well-being across the life span. It emphasizes the importance of combining multiple disciplines and corporate partners to produce a multifaceted cost-effective program that increases physical activity at all levels. The goal of this paper is to recognize exercise as a powerful, low-cost solution with positive benefits to cognitive, emotional, and physical health. Further, the model proposes that people of all ages should incorporate the “right to move” into their life style, thereby maximizing the potential to maintain health and well-being in a cost-effective, optimally influential manner.

## 1. Introduction

**Statement of the Problem**

The United States and much of the developed world is benefiting from increased longevity [1]. People are living longer. For the most part this longevity was, at least originally, accompanied by improvements in health and well being [1]. However, recent evidence clearly indicates a reduction in health and increases in chronic illnesses [2], many of which are exacerbated by, if not caused by, obesity, functional impairments, and disabilities [3–6]. The repercussions of these developments are significant in terms of an individual's quality of life [7], increased health care costs [2], and losses in productivity of the individual [8] and society [9]. Changing, that is, increasing, physical activity levels is very likely the most cost-effective and impressively effective intervention possible. Exercise is a powerful, low-cost solution with positive benefits demonstrated to effect cognitive, emotional, and physical health in people of all ages. However, rates of exercise have been declining over time [10]. As is evident, exercise is only effective to the extent that people actually do it.

**Trends in Physical Activity: A Dilemma with Health and Economic Consequences**

For several decades, governments and health departments around the world have embraced recommendations for “active aging” and the importance of physical activity at all ages for health and well-being [12]. Indeed, the US Department of Health and Human Services established goals in 2000 identifying physical activity as a leading health indicator and establishing goals to improve levels of activity by 2010 [13]. The Healthy People report suggested, for example, that by 2010, at least 25% of adults should report that trips of less than 1 mile were made by walking and that 30% of adults engaged in at least 30-minutes of moderate physical activity five days a week. Some may be astonished by these relatively low expectations, especially given decades of epidemiological and preventative medical research documenting the health benefits of lifestyles associated with regular physical activity and moderate exercise. However, these objectives actually reflect desperate calls to reverse long-term trends of increasing levels of inactivity and sedentary lifestyles that are predicted to have dire consequences on the U.S. economy population health and well being by 2030.

Cohort surveys and longitudinal panel data illustrate the changing trends in participation in *physical activity* (i.e., body movement linked to energy expenditure) and specific participation in planned or structured *exercise activities *done to improve or maintain physical fitness [10]. Respondents are typically asked about frequency of participation in leisure activity, household and yard work activity, occupational activity, self-powered transport (e.g., walking, cycling), and sedentary activity (e.g., sitting). National time-use diary surveys collect detailed information about the time allocated throughout the day to physical activities. Brownson et al. [10] reviewed time trends for these heterogeneous indicators of physical activity and found differential patterns. For example, nationally representative data collected in the Behavioral Risk Factor Surveillance System (BRFSS), revealed slight improvements for both men and women in levels of recommended physical activity (i.e., at least 30-minutes of moderate physical activity five days a week) from under 25% prevalence in 1990 to slightly over 25% prevalence in 2000. However, analyses of the Current Population Survey reveal that on average occupation-related physical activity levels have declined since the 1950s. With each decade, participation in low-activity occupations increases. Furthermore, the means of transport used to travel to work has changed considerably from the 1950s with the large majority driving (88% in 2000) rather than walking or using public transportation. These work-travel trends reflect substantial increases in associated time spent in sedentary activity (sitting) on daily commutes. Time-use surveys also document the increase in time spent in other sedentary activities from 1965 to 1995 (e.g., watching TV, using a computer) and the decrease in time spent doing housework (low level physical activity [14]). 

Multiple factors are associated with these differential trends in physical activity. The associations are complex and dependent on the type of activity examined. For example, whereas more highly educated people and those of higher socioeconomic status (SES) tend currently to participate more regularly in planned exercise programs, these same subgroups of the population are also more likely to be employed in sedentary occupations and to drive to work. The extent to which the overall time budget of this socially advantaged group reflects sufficient levels of physical exercise will also depend on work hours, availability in the local environment, job culture, and lifelong preferences for an active lifestyle. 

Talbot et al. [15] reported secular trends in leisure-time activities in relatively high SES and health conscious men and women in the Baltimore Longitudinal Study of Aging (BLSA). Overall time spent in leisure-time activity was relatively stable from 1960 to 1990. There was an average 10-min per day increase in high-intensity activity from 1960 to 1970 for men only but subsequent decades showed only marginal increases in this category. In the same period, the BLSA men were on average slightly overweight in all four decades of the longitudinal study and women, although initially not in this category in the 1970s had entered it in the 1990s. 

These population trends and the specific findings from a positively-selected group, who in general are expected to favor and adopt preventative health and active aging programs, highlight the extent and breadth of the overall dilemma. Indeed, a recent review of efforts to increase population levels of physical activity concluded that public health and intervention programs shift focus from recommending “moderate” levels of activity (e.g., at least 30 minutes of walking) to recommending change from zero activity to at least a low level [16]. 

The emerging problem and recommended solution, therefore, is to offset the increasing lack of physical activity by designing effective interventions that will increase the physical activity levels in people of all ages.

**Sustainable Increases in Physical Activity within the U.S. Population**

While there is clearly merit in inducing individuals of all age groups to increase physical activity and to feel personally accountable for their own health status, most experts believe that this is unlikely to gain widespread adoption in the near future. The quickest and clearest path toward universal change will be via organizations that are first responding to issues of their own economic well-being. These organizational platforms can then serve as the basis for individual and familial adoption of necessary lifestyle changes.

Companies and governmental and not-for-profit organizations that employ people are deeply concerned about the ongoing and future health status of both their employees and the families they support. Their concern derives from two perspectives. Healthy employees are clearly on the job more often (presenteeism), are more productive, and contribute to organizational life in ways their less healthy counterparts cannot. Ill family members clearly distract from the potential contributions an employee might make. And, most importantly, whether an organization is self-insured or pays for health care premiums via one of the numerous options, a healthy employee base will significantly moderate the costs associated with providing coverage.

Over the last 5–10 years many employing organizations have begun to collect detailed health status information from each of their employees (BP, LDL, HDL, height, weight, smoking status, alcohol use, exercise activity levels, etc.) through voluntary submissions, often monetarily incented. Some firms have taken steps to make the submission of this type of information mandatory while others have prohibited employees from engaging in certain behaviors that have known deleterious consequences to health. We may see this transition into programs that include all members of the family or household who are covered under the organizational or work place medical plan. 

The natural extension of these employing organizations concerned with productivity and health care cost issues is the implementation of programs that increase physical activity on either a voluntary and/or incented basis. Programs that induce and reward the expected behaviors will more quickly lead to the desired outcomes for employees and provide measureable outcomes that can be utilized to further adapt and adjust program parameters and performance. The assumption often made is that once employees are induced to higher levels of health performance, they will influence family members to model their behavior and increase their own health performance. From a marketing perspective, it can be assumed that organizations, industries, or corporations which actively market products and services that incentivize physical activity, will be quickly emulated by all others, especially as positive effects are demonstrated. Clearly influence can be seen, and hopefully understood and galvanized, at different levels from individuals who are personally motivated to improve their own health and perhaps influence their family members to engage in healthier lifestyles, to employers and societies interested in a healthier and more productive workforce. We turn next to a conceptual model designed to synthesize multiple levels of intervention within a single holistic and dynamic framework that has as its goal the increase of physical activity.

**A Conceptual Model to Address Physical Activity Deficit**

As outlined above, there is a vast literature on the importance of exercise for the individual and society, and on how to increase physical activity in people. What is missing from this literature is a larger conceptual framework that examines this problem at multiple levels and spheres of influence, and also examines how each level dynamically influences each other. We propose such a framework as a means to theoretically enrich our understanding of the embeddedness and complexity of physical activity inputs and to practically provide useful intervention points and intersections.

**Figure 1:**
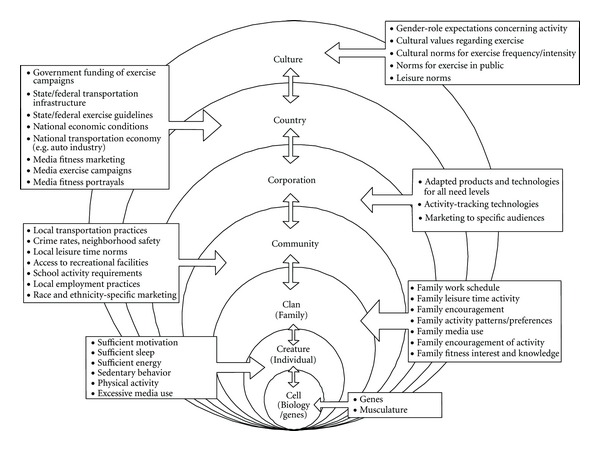
The 7-Cs Ecological Model of Physical Activity Across the Lifespan (Adapted from [11]).

In this paper we draw on an ecological systems framework [17] to address the issue of increased physical activity. In particular, we adapt a recently published ecological model on the childhood obesity epidemic [11] to address the issue of how to increase physical activity across the lifespan (see Figure 1 for our adapted model). Harrison and colleagues [11] present an ecological model that describes causes and potential intervention points of childhood obesity at multiple levels of analysis, ranging from genetic characteristics of the individual to environmental influences including proximal influences such as the immediate family environment to more distal influences such as the child's overarching cultural environments. Their 6-Cs ecological model includes the following spheres or levels.

1. Cell: this includes biological and genetic predispositions that might influence a child's likelihood of becoming obese. We adapt this level to apply to physical activity predispositions specifically.
2. Child: this level examines personal, psychological, and behavioral correlates of childhood obesity. We again adapt this level to apply to physical activity and re-label it “Creature” (or individual) to reflect our lifespan developmental approach that extends beyond childhood.
3. Clan: this includes family characteristics, processes, and dynamics, which we again apply only to physical activity levels.
4. Community: This level includes individuals' social worlds outside of their homes and may include their place of employment, school, religious organization, and any other community organization.
5. Country: institutional influences at the state and national levels are included at the country level of analysis, including national economic situations, government exercise guidelines, and media portrayals.
6. Culture: the culture or society level includes “culture-specific norms, myths, and biases that guide citizens' and policy makers' fundamental assumptions about exercise” [11, page. 51].

Although we rely heavily on Harrison et al.'s [11] model and theorizing, we make three adjustments in the current framework. First, we focus our theorizing exclusively on physical activity specifically, rather than obesity more generally. Next, we see this model as not only applying to children, but to individuals across the entire lifespan. Finally, we add what we believe is an important additional level to this model: Corporations (between the community and country levels; see Figure 1). We do so to specifically advocate for academic-industry partnerships in addressing the important issue of how to increase activity as people age. 

 It is corporations/organizations that play a dual role in promoting the right to move. They have the resources to act as a catalyst for their employees to embrace healthy lifestyles. They can also bring products and services to market that encourage the increased activity levels necessary to reduce obesity and enhance cognitive functioning.

**An Application of the Model: The Society 2030 Interdisciplinary Workgroup**

The above model was developed as part of a unique interdisciplinary workgroup, named Society 2030. The group is focused on meeting the demographic challenges that society will be facing in 2030 and beyond. Its goal is to review available data; outline, propose and/or conduct cutting edge research; and stimulate innovative solutions to problems resulting from the newly emerging society of the future. Society 2030 includes university and industry representatives who, through the cross-fertilization of ideas across disciplines and industries, are constructing a roadmap for the needs of Society 2030—based upon its structure, strengths, weaknesses and opportunities. Healthy life styles across the life span is a central theme and goal of the group.

Below we outline the usefulness of working within a multidisciplinary academic-industry conceptual framework to increase physical activity across the lifespan. Areas of expertise from several members of our workgroup are briefly outlined and placed within the larger conceptual model to address the physical activity deficit. These sections are intended to demonstrate the potential power of the intersectionality and triangulation that can evolve from the inclusion of such diverse perspectives. We emphasize, in particular, the importance of involving corporate partners in future initiatives to increase active lifestyles. We highlight several (but not all) levels of the conceptual model (Figure 1), and seek to integrate these divergent perspectives into innovative solutions that will effectively increase physical activity among multiple population groups.

## 2. Creature (Individual)

At the individual level an important related effect of physical activity is the cognitive functions that are necessary for successful mobility. Changes that occur with aging (and disease) that affect one's ability to perform motor tasks may have a limiting effect on the physical activity in which an individual can engage. Tasks such as balancing (referred to as postural maintenance) and walking have been shown to require cognitive processes such as attention, response selection, and mental flexibility [18, 19]. The ability to do two things at once, known as dual tasking, is particularly important for activities of daily living, such as walking while holding groceries, or having a conversation on a cell phone. The consequences of not being able to do two things at once, for example, could lead an individual to focus only on talking on the phone, and put that person at a much higher risk of falling as he/she would not be able to notice and/or react to an upcoming obstacle or a change in the pavement.

 As these types of cognitive skills have been shown to become less efficient with age, older adults have more difficulties with dual tasking. Studies have shown that this age group is at higher risk of tripping and falling [20, 21]. In addition, even after controlling for factors such as medical health and physical changes, individuals with documented cognitive impairment have been shown to have more problems with activities such as postural maintenance and ambulation, especially under dual task conditions. This puts them at a much higher falls risk [22–24]. These findings highlight the need for designers of mobility/exercise programs to understand the role of cognitive functioning in mobility. This is an important factor in the design of appropriate interventions that consider the cognitive load associated with any task as well as potential age and disease related cognitive changes that can impact whether an individual can benefit from the program. 

Exercise is not only dependent on cognitive abilities, but has also been shown to have a beneficial effect on cognitive functioning in young and old adults. Those who engage in regular physical activity demonstrate better performance on a range of cognitive measures including memory, attention, information processing speed, response time, and tasks such as decision making and problem solving (often referred to as executive functions) [25–28]. These findings have been shown with a variety of exercise programs, with some suggestion that exercise programs that target aerobic efficiency may be somewhat more beneficial to cognitive functioning [29–31]. Exercise related cognitive improvements have also been demonstrated in individuals with definite cognitive impairment. A recent meta-analysis suggested that exercise training can improve cognitive functioning in individuals with Alzheimer's disease and related dementias [32], while there is also some suggestion that exercise may reduce the risk of dementia in older adults. Sumic and colleagues [33] found that older women (in the “oldest-old” age category) who engage in physical activity show an 88% risk reduction of cognitive impairment compared to inactive women. 

Although exercise can be a solitary activity, often people plan and engage in such activities with others, thereby increasing their social activities and enhancing the quality of their social relations. The benefits of positive and supportive relationships on health and well-being have been well documented [34]. In particular, Convoys of Social Relations can play an influential role in persuading individuals to improve their health behaviors [35–38]. For example, although there is a longstanding awareness of the positive effects of high quality social relations on health [39], recent more nuanced research has documented that the negative aspects of relationships (i.e., get on nerves; demanding) are associated with greater longevity [40, 41]. This suggests that negative relationship quality although linked to greater stress, may also play some role in facilitating and sustaining health promoting lifestyle and behavior changes [42]. 

## 3. Community

There is promising new research that best fits into the Community level of our conceptual framework (Figure 1) and can help address the physical activity deficit in a way that capitalizes on fundamental human needs for social connection and interaction [43]. Researchers have long known that social relationships have positive effects on later health outcomes that are at least as robust as more traditional health risk factors such as smoking, high blood pressure, and even physical activity levels [44]. In fact, a recent meta-analysis finds that those with strong social relationships have a 50% increase in the likelihood of survival, even when taking other risk factors into account [45]. 

Of particular interest is how regularly structured social activities within communities, such as religious service attendance and volunteering, affect health across the lifespan, especially among older adults, who are known to have relatively high levels of both religious and volunteer participation [46–48]. Much research has found evidence for the physical health benefits of both religious attendance [49, 50] and volunteering [51]. It is believed that both of these types of structured social activities positively influence the physical health of older adults via psychological (e.g., increased meaning and purpose in life) and biological mechanisms (e.g., better stress regulation, improved immune functioning); [49, 51].

At the same time emerging perspectives are providing evidence that one additional way that religious activities and volunteering behaviors could ultimately influence long-term physical health among older adults is by increasing their levels of physical activity. Although no research which we are aware points to increased activity levels as the sole mechanism responsible for explaining health benefits, there are research findings indicating that older adults who are actively religious have higher levels of physical activity than those who are not [52, 53]. Similar outcomes are present among those who are active volunteers. In fact, field experiments that assign older adults to intensive volunteering (e.g., Experience Corps) find significant increases in physical activity levels after the volunteering experiences begin [54, 55]. Taken together, it is not surprising that activities that get people out of the house make them more physically active, but such activities are especially promising because they rely on *social* motivations to get moving rather than the elusive intrinsic desire to simply exercise more. 

Importantly, although religious and volunteer participation both fit into the Community level of analysis, these behaviors are most likely to be effectively channeled into increased physical activity to the extent that they are considered at multiple levels of our conceptual model. For example, at the Creature (or individual) level, individuals who are already feeling healthy will be more likely to both attend religious services and volunteer [56, 57]. At the Clan (or family) level, it is more likely that older adults will choose to get out of the house and participate in such activities if they are encouraged, valued, and engaged in by other members of their family. And at the Corporation level, increased religious and volunteer participation is likely to be facilitated by products that support the unique physical needs of an aging population (e.g., comfortable shoes adapted to older people so that they can be on their feet for longer each day). 

Another aspect of the Community that can have an impact on physical activity levels is the built or physical elements of the environment. This could include worksite exercise facilities, bike paths, walking trails, gyms, swimming pools, sidewalks, and nice scenery. It is not surprising though that communities with greater financial resources have been documented to have greater access to these types of physical activity encouraging resources [58–60]. 

## 4. Corporations

Corporations generally develop products and services that respond to market demands. In the future corporations and other organizations should take a more proactive approach that drives the market in the direction of recognizing the right to move and encourages healthier life styles.

**Figure 2:**
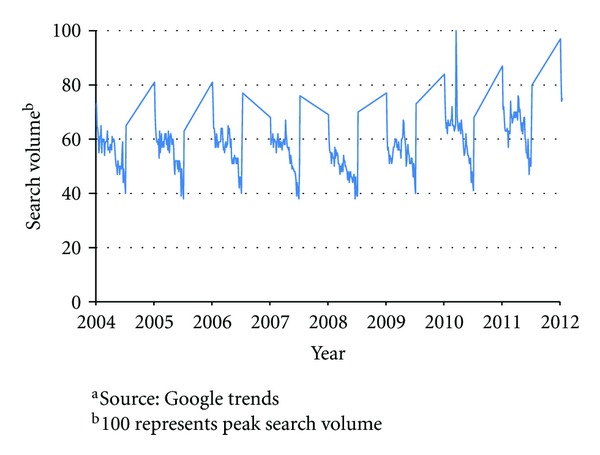
Web Search Interest in “Weight Loss” (2004–present). Source: Google trends.

Lack of physical activity is resulting in a variety of health challenges, which is certainly driving public and corporate awareness of this important issue. If we examine, for example, through Google search, trends between 2004 and the present, an increasing trend of news coverage for “weight loss” is evident (see Figure 2). In addition, consumer searches for “weight loss” have shown a clear shift starting in 2010 following years of consistent patterns and recorded solid growth for past two years. 

The current environment indicates increased public awareness and consumer demand for “weight loss.” Nevertheless, consumer's engagement with physical activity has seen some setbacks and CDC reports only a slight decline in people reporting no leisure time physical activity over the last twenty years [61]. This combination of developments suggests that individuals preferred path towards weight loss may not always be through physical activity.

Industry has been fairly receptive to rising consumer awareness of weight loss. Many companies are offering an increased selection of related products in a variety of categories. For example, in recent years food and beverage products are increasingly advertising known health benefits such as “No Trans Fat,” “High Fiber/Whole Grain,” “100% Real Fruit,” “Low Salt/Sodium,” and “Natural/Organic.” However, the trend towards internet shopping and super stores while providing the convenience of one-stop shopping also reduces the physical activity necessary for daily activities with parallel increases in morbidity and mortality.

An interesting new development has focused on the promise of smart clothes and shoes in helping to maintain a physically active lifestyle across the lifespan. The advent of mobile computing, miniaturized electronics, and ubiquitous Wi-Fi has introduced the possibility for individuals of any age to wear clothing, shoes, and head bands or hats that continuously measure physical activity, body weight, and physiological signals safely and non-invasively. This is as true for the neonatal infant, as it is for the professional athlete and the frailest of nursing home residents. Body-worn sensors can be networked via fine wires sewn into the fabric of clothing, or conductive pathways coating certain threads in textiles. In addition, body-worn sensor networks can be linked wirelessly to a PDA worn by the individual, or via Wi-Fi, to a central recording location for computer and data storage facility where it is needed. These modern instruments and products can serve to encourage a healthier lifestyle.

Physical activity can already be monitored using miniature inertial measurement units (IMU) that continuously measure the linear accelerations and angular velocities of each body segment. A magnetometer in the IMU continuously tracks the posture of one or more body segments with respect to gravity, so one can ascertain whether an individual is sleeping, sitting, standing, or locomoting. A GPS sensor can track horizontal and vertical distance moved to check the distance walked or run for exercise. Other sensors can non-invasively track heart rate, body temperature, muscle activity, body weight via the pressure under the feet when standing, pressures on the skin of a vulnerable body part to prevent ulcers while sitting or sleeping, the number of movements during sleep to evaluate sleep quality, the excess tension on the waist belt used to keep trousers up due to inadvertent adiposity, inadvertent urine loss for those with incontinence, falls in the elderly, concussion in the athlete, and even the activity of surface networks in the brain. Sensors are already so small and inexpensive that they can even be placed in food to monitor gastric health and vital signs internally, after which they are discarded with body waste. By the year 2030 it is safe to assume that the routine use of body worn, and likely also implanted, sensor networks, will routinely be used to monitor all the physiological signs needed to optimize health both when awake and asleep.

The use of biofeedback from these sensors will allow an individual, family member, trainer, coach or physician to monitor activity levels and determine whether they are less than adequate, optimal, or have reached a level that they are likely to be injurious due to overuse. Social networking is already allowing individuals to compare physical activity logs, and this is likely to become increasingly widespread, potentially serving as an important incentive to engage in physical activity. The use of miniature body-worn actuators, such as vibrators, mounted in clothing already allows users to receive feedback on negative or positive behaviors, to learn new skills via knowledge of results, or relearn old skills like walking during a rehabilitation program. They can even be used to warn drowsy drivers when their behavior has become dangerous to themselves and others.

As this brief overview indicates, there are numerous developments at the product or corporate level which can positively or negatively influence physical activity. As an example, when coupled with incentives, the development of inexpensive “smart” clothing and body-worn instrumentation holds a great deal of promise for reducing the negative while fostering behaviors and behavior change consistent with a healthy lifestyle across the lifespan. 

## 5. Country

Insights from the brain may help predict responsiveness to public efforts to proactively influence behavior. Mass media representations of people being active (or sedentary) may be one important way in which norms around physical activity develop. As such, an understanding of media effects is essential to traversing levels of analysis from culture to creature (Figure 1). Important sources of mediated social influence include public service announcements, representations of activity norms in entertainment media, and other forms of persuasive messaging (e.g., advertisements). However, not all messages are equally effective in motivating behavior change, and not all individuals are equally affected by messages promoting healthy changes. 

 Recent neuroimaging research suggests that the brain may offer insights about the potential success of media campaigns, above and beyond people's self-reports. For example, neural responses to public service announcements promoting sunscreen use [62] and smoking [63] predict individual behavior change in the weeks and months following the scan. Furthermore, these neural data explain variance in behavior change above and beyond individual participants' reports of their intentions to change their behavior, their attitudes about the behavior, their confidence in their ability to change their behavior, and their ability to relate to the advertisements. Likewise, neural activity may also help scientists and practitioners select the best messages and interventions to motivate the largest number of people. Smokers' neural activity in response to advertising campaigns promoting the National Cancer Institute's tobacco quitline correctly predicted the success of the different campaigns in increasing quitline call volume at the population level, whereas the participant's self-reports of which advertisements they thought would be most effective did not [64]. Researchers have also demonstrated the utility of neuroimaging in predicting the popularity of other types of media [65]. Such methods have not been applied to predict the success of messages or interventions targeting physical activity, but these methods may be applied to design messages and interventions to bend, that is, improve, the health care cost curve over the next several decades.

Finally, the brain can aid in our understanding of the basic mechanisms that lead people to make healthy decisions, and to successfully change their behavior toward healthier habits. For example, cognitive control activity in the brain during a response inhibition task predicts smokers' ability to break the link between craving and smoking [66], and activity in the brain's reward network in response to food stimuli predicts changes in body mass index over the months following the scan [67]. This brain-as-predictor approach [68] may also be key in developing our understanding of the mechanisms that lead people to be successful in increasing their physical activity.

Social Media, defined as “any online platform or channel for user generated content” [69] includes networking portals such as Facebook, MySpace, and LinkedIn; instant messaging systems such as Twitter; online video-sharing websites such as YouTube; photo-sharing websites such as Flickr; and blogging platforms such as Tumblr, among many others [70]. Social media platforms and technologies have evolved dramatically, and have become nearly ubiquitous given their popularity among younger cohorts. American adolescents are actively engaging with peers through social media outlets, with studies reporting that they spend more than 2 hours per day on the internet and on average, 80% of that time is spent on a social network [71]. In addition to their growing use of social media, adolescents today are also more likely to carry a cell phone than any previous generation. In fact, mobile phones are nearly ubiquitous among U.S. teens, with three-quarters of them reportedly owning a mobile phone in 2010 [72]. This trend appears to be worldwide.

 Obesity prevalence among U.S. children, defined as BMI ≥ 95th percentile for age and sex, has increased dramatically, with a greater than 3-fold rise since 1976—rising from approximately 5% to 17% [4, 73]. Despite evidence that physical activity could, in part, mitigate this dire trend, studies show that U.S. adolescents are not getting sufficient exercise [74]. Only about one-half of U.S. adolescents reportedly participated in vigorous physical activity on a regular basis in 1996; one-fourth reported no vigorous physical activity at all [74]. Since the adolescents of today are the middle-aged adults of 2030, these trends do not bode well for the health and well-being of our society in 2030. Given these trends, there is a growing interest among public health professionals to leverage social media platforms to incentivize adolescents to engage in healthy behaviors. The effectiveness of these tactics would likely hinge on the proven effects of social networks and peer groups on individual activity. Studies have shown that physical activity levels among adolescent females are enhanced by having friends who are more physically active [75], and that positive feedback is also an important element or factor contributing to enhanced levels of physical activity [76–79]. Social media and mobile technology therefore have the potential to leverage these effects as they relate to levels of adolescent physical activity. 

 There is evidence from the literature that social media and mobile platforms can be used to successfully promote physical activity. One recent study of U.S. college students found that a weight-loss program that was administered to participants through Facebook and Twitter was indeed effective [80]. In another recent study of overweight adults, researchers found that text-messaging was a productive means of promoting behaviors supporting weight loss [81]. These results are promising. Based on this trend, there is an increasing number of social media applications geared towards fitness and overall health—particularly those with mobile accessibility—that have the potential to mitigate low levels of physical activity. 

 Fitocracy and MapMyFITNESS are two examples of fitness social networks, accessible through online platforms that connect users who are devoted to exercise. These sites allow users to log workouts, follow friends' workouts, count calories burned, and be awarded points for achieving fitness goals. By leveraging the productive influences of social and peer engagement, these platforms and others like them may serve to incentivize users to increase their physical activity and to live healthier lives. 

 Gaming is another emerging phenomenon on social media and mobile platforms. The Pew Research Center study reported in 2010 that 46% of teen cell phone owners played games on their mobile phones [82]. A second study conducted by ROIWORLD, an online gaming site, found that much of the time spent by teens on social network sites was devoted to playing video games. In fact, gamers on Facebook and MySpace reported spending 6+ hours per week playing games [71]. Gaming has been implicated as a potential determinant of the childhood obesity epidemic, but researchers are now leveraging gaming technology to help combat the epidemic. A number of studies have evaluated the effectiveness of using video games to increase physical activity [83, 84]. Some studies have found that active games like Nintendo Wii's dance, dance revolution (DDR) could improve physical fitness in children [85–87], and in one instance provides a more vigorous workout compared with walking on a treadmill at 3 miles per hour [85]. Location based games are another creative avenue for increasing physical activity. SCVNGR is a mobile application available to iPhone and Android users, which provides location based alerts through the GPS on the phone. This allows users to devise challenges or games at specified locations, select a competitor for the challenge among their social network, and earn points once those challenges are completed. SCVNGR could be used as a game to encourage physical activity across a geographic space, by having adolescents walk to different physical locations, motivated by the games or challenges and their social network of friends/competitors. The potential of mobile and social media platforms for improving physical activity levels among youth, and possibly adults of all ages, are endless. 

 Despite these promising developments a note of caution should be raised. Exercise interventions and guidelines should not be excessive or raise unrealistic expectations. Since recent evidence [16] has shown that even mild to moderate amounts of physical activity can have significant health benefits, all levels of activity should be recognized and applauded.

## 6. Future Directions and Concluding Remarks

 In this paper we highlight the alarming trend of inactivity among children, adolescents, young, middle, and older people. We summarize evidence indicating that growing trends of increased obesity, related illnesses and disabilities will result, by 2030, in a population that is heavier, sicker, and more disabled, and a society that is overburdened with health costs and is considerably less productive. We urge a proactive approach which benefits from the well known significant and positive effects of physical activity and outline methods to encourage this low cost, pervasively effective health behavior. We have presented a multidimensional theoretical framework, ranging from cells to culture, that focuses on the intersectionality of those factors influencing physical activity across the lifespan. We highlight specific intervention possibilities of select dimensions of the model. 

Further, we posit there are direct effects the corporation/organization (as an employer) has directly on its employees and family through an architecture of positive health behaviors that are either forced or incented. The longer-term effects are increased positive health for employees and those familial members touched by the health care coverage umbrella. These same corporations/organizations have indirect effects on the communities and country/culture in which they operate through their messaging about the right to move and the importance of improved physical activity on the part of their employees and families. They may also use messaging (and sponsorships) toward their customers to engage in healthier lifestyles via products/services that reinforce the central theme.

We further emphasize the importance of working within multidisciplinary teams that include academic researchers and corporate partners who will recognize the importance of incentivizing physical activities and of empowering individuals with the right to move, thereby optimizing the potential of health and well-being across the life-span, especially through active aging.
